# Injectable human tendon extracellular matrix via isopropanol-assisted delipidation and mild decellularization for tendon regeneration

**DOI:** 10.3389/fbioe.2026.1745558

**Published:** 2026-07-07

**Authors:** Jung Ki Lee, Jiyeon Mun, Hee Yeon Kim, Yeon-Seop Jung, Sang-Hyun An, Changsoo Kim, Kee-Won Lee

**Affiliations:** 1 R&D Center, L&C BIO Co., Ltd., Seoul, Republic of Korea; 2 Preclinical Research Center, Daegu-Gyeongbuk Medical Innovation Foundation (K-MEDI hub), Daegu, Republic of Korea

**Keywords:** human tendon ECM, injectable biomaterial, isopropanol-assisted delipidation, mild decellularization, tendon injury, tendon regeneration

## Abstract

Tendon injuries remain a major clinical challenge due to their limited intrinsic healing capacity and the shortcomings of current treatment options. Extracellular matrix (ECM)-based approaches are promising; however, most clinically available injectable ECMs are xenogeneic, raising concerns about immunogenicity, pathogen transmission, and limited durability. Human-derived injectable ECMs have recently gained attention, yet conventional solubilization-based preparations often disrupt native architecture and compromise biological function. Here, we present an injectable decellularized human tendon (DHT) produced by a dual-step isopropanol (IPA)-assisted delipidation combined with mild decellularization. This process effectively removed lipids and cells while preserving ultrastructure, collagen organization, and key growth factors, with no cytotoxic residues detected. Notably, instead of chemical solubilization, injectability was achieved by micropulverization and hydration, enabling minimally invasive delivery while retaining structural integrity and native ECM cues. *In vitro* studies demonstrated that injectable DHT supported the viability and proliferation of human tenocytes and promoted tenogenic differentiation of human adipose-derived stem cells. In a rat Achilles tendon injury model, injectable DHT significantly enhanced ECM remodeling, neovascularization, and functional tendon regeneration compared with human acellular dermal matrix and porcine collagen controls. For the first time, this study demonstrates that an IPA-assisted, mildly decellularized, and mechanically processed human tendon ECM can be formulated into an injectable while preserving its native architecture and bioactivity. To our knowledge, this study presents one of the first injectable, human tendon–derived ECM formulations specifically designed as a flowable, tissue-specific therapeutic platform for tendon regeneration, and systematically evaluated for its biological performance both *in vitro* and *in vivo*.

## 1 Introduction

Tendon injuries are common, often presenting as tendinitis or tendinosis, and frequently result in significant functional impairment (Yang et al., 2013; Chartier et al., 2021; Citro et al., 2023). The annual incidence of Achilles tendon ruptures ranges from 7 to 40 cases per 100,000 person-years, with a notable increase over the past three decades (Lantto et al., 2015; Ann et al., 2016). Reflecting this burden, the global tendon repair market was valued at USD 2.3 billion in 2023 and is projected to reach USD 4.0 billion by 2030 (Research, 2024).

Tendon-to-bone and tendon-to-ligament interfaces are mechanically complex, obscuring complete recovery (Doschak and Zernicke, 2005). Acute overload induces inflammatory tendinitis, whereas chronic degeneration leads to tendinosis and loss of strength (Arvind and Huang, 2021). Conventional treatments, including rest, ice, compression, elevation (RICE) and physical therapy, are known to relieve symptoms, however, they have limited efficacy in chronic cases, and repeated use of corticosteroid injection may lead to clinical adverse effects including weakening tendons (Sussmilch-Leitch et al., 2012; Čobec and Kozinc, 2022). Surgical interventions are reserved for refractory cases but carry risks of infection, scarring, and biomechanical compromise (Abbas et al., 2022). Autologous tendon grafts are generally free of immune rejection but require healthy donor tissue and are unsuitable for bilateral or systemic degeneration (Biau et al., 2006; Widner et al., 2019; Nikumbh et al., 2024).

To address these limitations of current treatments, the platelet-rich plasma (PRP) or mesenchymal stem cell (MSC) based applications have been explored as injectable therapies for tendon repair (M'Cloud et al., 2024; Rahman et al., 2024). However, clinical outcomes remain inconsistent due to the variability in preparation protocols and donor-dependent factors. These challenges ignite the need for alternative injectable approaches with more predictable efficacy. Tissue-derived extracellular matrix (ECM) biomaterials represent a promising option, as they can be locally delivered to degenerative tendon tissue minimizing the risks associated with surgery.

Among biomaterial-based strategies, the decellularized tendon ECM has increasingly gained attention for its ability to promote cell infiltration and modulate immune responses (Schulze-Tanzil et al., 2012; Kim et al., 2024). Tendon ECM retains bioactive factors, including transforming growth factor beta (TGF-*β*), vascular endothelial growth factor (VEGF), and basic fibroblast growth factor (bFGF), that play critical roles in the cellular regulation and the tissue remodeling (Molloy et al., 2006; Lin et al., 2023). While most studies have utilized xenogeneic sources such as porcine or bovine tendon, (Alberti and Xu, 2016; Huang et al., 2023) concerns still remain regarding immunogenicity, zoonotic pathogen transmission, and long-term structural stability (Al-Hakim Khalak et al., 2022; Russo et al., 2022). In contrast, the decellularized human tendon (DHT) offers superior biocompatibility and preserves native ECM architecture, rendering it a safer and more clinically relevant alternative (Kasravi et al., 2023; Runer et al., 2023). Its ultrastructural and biochemical integrity supports the tenocyte repopulation, neovascularization, and the restoration of mechanical properties, all essential for functional regeneration (Schulze-Tanzil et al., 2012; Runer et al., 2023). Previous studies to develop injectable DHT for minimally invasive delivery, (Farnebo et al., 2014; Farnebo et al., 2017; Kaizawa et al., 2019) have typically relied on the complete ECM solubilization using acid treatments (Seif-Naraghi et al., 2012; Kim et al., 2016; Jiang et al., 2021; Li et al., 2024). Such processing inevitably disrupts native tendon architecture and biochemical cues, thereby diminishing the regenerative potential of tendon ECM when converted into fully solubilized injectable forms.

Although decellularization efficiently removes the cellular components from human tendon ECM, (Moffat et al., 2022) recent evidence indicates that the residual lipids can act as pro-inflammatory and immunogenic agents, particularly in the scaffolds derived from human tissues (Yang et al., 2020). Such lipid remnants may elicit host immune responses and compromise both biocompatibility and regenerative efficacy, emphasizing the importance of effective delipidation for clinical safety (Song et al., 2018; Ventura et al., 2019; Ahn et al., 2022). Aggressive solvent systems such as chloroform–methanol, although effective for lipid removal, may compromise ECM integrity, whereas alcohol-based solvents are considered relatively mild and more compatible with ECM preservation (Crapo et al., 2011). Consistent with this principle, IPA-assisted decellularization has been shown to enable efficient delipidation while maintaining ECM structure (Ventura et al., 2019), supporting its selection for injectable DHT development.

In this study, we aimed to develop an injectable DHT as a novel strategy for tendon regeneration. While most injectable ECM products are xenogeneic, to the best of our knowledge, no human tendon-derived injectable ECM has been commercially approved or clinically translated to date, likely due to the limited tissue yield and the processing challenges (Molloy et al., 2006; Kasravi et al., 2023; Lin et al., 2023; Runer et al., 2023). Previous studies using human tendon-derived ECM have mainly employed enzymatic solubilization followed by hydrogel reconstitution, in which injectability relies on pre-gel solutions and subsequent *in situ* gelation, often accompanied by handling complexity and limited clinical practicality (Molloy et al., 2006; Farnebo et al., 2014).

To address these issues, in the present work, we combined mild decellularization with IPA-assisted delipidation to remove cells and lipids while preserving ECM integrity and key growth factors. Instead of full solubilization, we adopted a particle-based strategy by mechanically fragmenting and hydrating decellularized human tendon ECM, achieving injectability through physical deformability and flow of hydrated ECM particles while retaining native fibrillar structure and tissue-specific biochemical cues. Accordingly, this formulation represents, to the best of our knowledge, a distinct injectable DHT that is mechanistically and translationally differentiated from previously reported solubilized or hydrogel-based systems. We comprehensively characterized the physicochemical and biochemical properties of DHT, including ECM composition, structural integrity, growth factor retention, and residual processing reagents. We further evaluated its biological performance through *in vitro* cytocompatibility and tenogenic differentiation at both gene and protein levels. In addition, we evaluated its *in vivo* therapeutic potential in a rat Achilles tendon injury model using immunohistochemistry (IHC), magnetic resonance imaging (MRI), functional assessment (Achilles Functional Index, AFI), histological analysis with Bonar scoring, and biomechanical testing. These multi-level evaluations demonstrate the potential of injectable DHT as a clinically translatable tendon-specific ECM therapy.

## 2 Materials and methods

In this study, the term DHT refers to decellularized human tendon ECM, while injectable DHT denotes the injectable formulation prepared by suspending micropulverized DHT particles in normal saline.

### 2.1 Preparation of human tendon tissue

Donated human tendon tissues were obtained from United States tissue banks in compliance with the regulations of the American Association of Tissue Banks (AATB) and the U.S. Food and Drug Administration (US FDA). Donor medical histories and serological test results were reviewed and approved by a certified medical director. Human tendon tissues were obtained from seven donors (5 males and 2 females) with an age range of 40–75 years. Donor demographic information, including sex and age, is summarized in Supplementary Table S1. All tendon tissues were processed using the same standardized protocol to minimize potential variability among donors. Fresh human cadaveric tendons were harvested, and surrounding soft tissues were carefully removed. To prevent dehydration prior to processing, the tendon tissues were immediately immersed in the sterile distilled water.

### 2.2 Optimization of decellularization and delipidation protocols for DHT preparation

*Preparation of DHT.* Tendon tissues were treated with antibiotic–antimycotic solution (Gibco, USA) for 30 min and were washed with distilled water. The results from three decellularization protocols were compared: (i) 1% SDS for 24 h, (ii) 0.5% SDS for 2 h, and (iii) 80% isopropanol (IPA) for 2 h followed by 0.5% SDS for 2 h. For delipidation, native tendon, 2-step (IPA + SDS), and 3-step (IPA + SDS + IPA) treatments were tested.

### 2.3 Characterization of DHT

#### 2.3.1 Histological analysis

Samples were fixed in 10% buffered formalin, embedded in OCT compound (Sakura Finetek, USA), and cryosectioned at 8 μm using a cryostat (CM 1950, Leica, Germany). Sections were stained with hematoxylin and eosin (H&E, Thermo Scientific, USA) and Oil Red O (Sigma-Aldrich, USA).

#### 2.3.2 DNA quantification

Lyophilized samples (10 mg) were digested with tissue lysis buffer (iNtRON Biotechnology, Korea) and proteinase K (Thermo Fisher Scientific, USA), followed by phenol–chloroform extraction. DNA contents were measured using the Quant-iT™ dsDNA Assay Kit (Thermo Fisher Scientific, USA; excitation/emission, 480/520 nm).

#### 2.3.3 Lipid quantification

Residual triglycerides were quantified using the Ez-Triglyceride Kit (DG-TGC100, Dogenbio, Korea). Dried samples (20 mg) were digested in 5% Triton X-100, incubated with lipase and were reacted with the probe solution. The absorbance was measured at 570 nm.

#### 2.3.4 Collagen quantification

Collagen contents were determined from the hydroxyproline assay. Acid-digested samples were reacted with chloramine T solution and Ehrlich’s reagent, and the absorbance was measured at 550 nm using a microplate reader (Varioskan LUX, Thermo Fisher Scientific, USA).

#### 2.3.5 SDS-PAGE

Lyophilized collagen samples were solubilized in 0.01 N acetic acid, were reduced with *β*-mercaptoethanol, and were separated on 7.5% SDS–PAGE gels. Protein concentrations were determined using the BCA assay (Thermo Fisher Scientific, USA). Gels were stained with Coomassie brilliant blue (Biosesang, Korea) and were destained in an acetic acid/methanol solution. *α*-, *β*-, and *γ*-chain bands have been analyzed to confirm the collagen integrity and purity.

#### 2.3.6 SEM observation

Freeze-dried samples were sectioned along the fibril alignment, were sputter-coated with gold (SPT-20, COXEM, Korea), and were imaged using a tabletop SEM (EM-30N, COXEM, Korea) to evaluate collagen fibrillar alignment and ECM ultrastructure.

### 2.4 Growth factor analysis of DHT

Cytokine and growth factor levels in supernatants from the native tendon and DHT have been measured using the Luminex Performance Human XL Cytokine Panel (9-Plex, LUXLM000B) and the Human TGF-β Panel (1-Plex, LTGM00) kits (R&D Systems, USA). The 9-Plex panel included IL-8/CXCL8, VEGF, PDGF-AB/BB, and PDGF-AA, while the TGFβ panel contained TGF-β1. Supernatants were centrifuged at 10,000 g for 10 min at 4 °C to remove debris and were stored at −80 °C until analysis. For the TGF-β1 measurements, samples were activated with 1 N HCl for 10 min at room temperature, were neutralized with 1.2 N NaOH/0.5 M HEPES, and were immediately assayed. Standards, controls, and 100 µL of each sample were added to the 96-well plates. The plates were then incubated at room temperature with gentle shaking according to the manufacturer’s protocols. The wells were washed, and the biotinylated detection antibodies were applied followed by the HRP-conjugated streptavidin. The color was developed using TMB substrate and was stopped with 1 N H_2_SO_4_. The absorbance was measured at 450 nm, and the cytokine concentrations were calculated from the standard curves generated with the recombinant standards provided in the kits.

### 2.5 Quantification of residual antibiotics, IPA and SDS in DHT

#### 2.5.1 Residual antibiotics

Residual antibiotics in the tendon tissues and DHT have been quantified using HPLC (600S controller, Waters, USA) with a Phenomenex Luna C18 column (150 mm × 4.6 mm, 5 µm). The mobile phase consisted of 100% acetonitrile and 0.1% formic acid in water at 1.0 mL/min and 30 °C. Detection was performed with a UV detector at 385 nm for amphotericin B, 240 nm for penicillin G, and 200 nm for streptomycin. Each sample was analyzed for 20 min, and the residual levels were reported in ppm.

#### 2.5.2 Residual IPA

Residual isopropanol (IPA) was measured using GC-FID (8890 GC system, Agilent, USA) with a DB-624 column (20 m × 0.18 mm, film thickness 1.0 µm). Nitrogen (N_2_) was used as the carrier gas at 1.0 mL/min. The injector and the FID temperatures were set to 220 °C and 250 °C, respectively. Each sample was analyzed for 10 min, and the residual IPA was quantified in ppm.

#### 2.5.3 Residual SDS

Residual SDS was determined by HPLC (1,260 Infinity II system, Agilent, USA) using a C18 column (150 mm × 4.6 mm, 5 µm). The mobile phase consisted of 100% acetonitrile and 5 mM ammonium acetate in acetonitrile:water (50:50, *v/v*) at 1.0 mL/min and 40 °C. Detection was performed with an evaporative light scattering detector (ELSD) at 35 °C. Each sample was analyzed for 10 min, and the residual SDS was reported in ppm.

### 2.6 *In vitro* assessment of the cellular responses to DHT

#### 2.6.1 Cytotoxicity

*In vitro* cytotoxicity was assessed according to ISO 10993-5. Mouse fibroblast cells (L929, Korean Cell Line Bank, Korea) were seeded in 6-well plates (2 × 10^5^ cells/well) in MEM (Biowest, France) supplemented with 10% FBS (Thermo Fisher Scientific, USA) and 1% penicillin–streptomycin (P/S, Thermo Fisher Scientific, USA). The polyethylene film and the zinc diethyldithiocarbamate (ZDEC) polyethylene film were used as negative and positive controls, respectively. The DHT extracts for cell assays were prepared according to ISO 10993-12 by incubating samples in MEM at 37 °C for 72 h, followed by filtration through 0.22 µm syringe filters. After 48 h, the cell morphology and the viability were evaluated using optical microscopy, and the regions of interest (ROIs) were analyzed using ImageJ to determine average cell counts.

#### 2.6.2 Cell culture, viability, and proliferation

Human tenocytes (Innoprot, Spain) were cultured in MEM with 10% FBS and 1% P/S at 37 °C with 5% CO_2_. The cells at ∼80% confluence were detached with 0.25% trypsin/EDTA (Thermo Fisher Scientific, USA) and were subcultured. The cell viability was assessed using the Live/Dead Viability/Cytotoxicity kit (Invitrogen, USA). The tenocytes were seeded on poly-L-lysine coated glass coverslips (1 × 10^4^ cells/cm^2^) and incubated with the DHT extract. After 1, 2, and 4 days, the cells were stained with calcein AM and ethidium homodimer-1 for 30 min at room temperature and were observed under a fluorescence microscope (Axio Imager M2, Zeiss, Germany). The cell proliferation was evaluated using the CCK-8 assay. The cells were seeded in 96-well plates (1 × 10^4^ cells/well), were treated with DHT extract, and were incubated for 1, 2, and 4 days. The absorbance at 450 nm was measured using a microplate reader.

### 2.7 Tenogenic differentiation of *h*ADSCs induced by DHT

#### 2.7.1 qRT-PCR

Human adipose-derived stem cells (*h*ADSCs, StemPro, Thermo Fisher Scientific, USA) at passage 5 were seeded with 1 × 10^4^ cells/cm^2^. When ∼80% confluent, the cells were cultured for up to 10 days in: (i) growth medium (GM) (α-MEM supplemented with 10% FBS and 1% P/S), (ii) differentiation medium (DM) (DMEM supplemented with 2% FBS, 1% P/S, 1% ITS-X, 1% ascorbic acid, and 10 ng/mL TGF-*β*3), or (iii) differentiation medium supplemented with the DHT extracts. The tendon-specific differentiation was evaluated by qRT-PCR. Total RNA was isolated on days 3, 7, and 10 using an RNeasy Mini Kit (Qiagen, Germany), and the first-strand cDNA was synthesized with iScript™ cDNA Synthesis Kit (Bio-Rad, USA). qRT-PCR was performed using SYBR Green Supermix (Applied Biosystems, USA) on a StepOne Plus system (Applied Biosystems, USA). Relative expressions of SCX, TNC, and TNMD were calculated using the ΔΔC_T method and were normalized to GAPDH. The primer sequences were as follows*:* SCX (F: 5′-ACA​CCC​AGC​CCA​AAC​AGA-3′, R: 5′-GCG​GTC​CTT​GCT​CAA​CTT​TC-3′); TNC (F: 5′- ATG​TCC​TCC​TGA​CAG​CCG​AGA​A -3′, R: 5′- AGT​CAC​GGT​GAG​GTT​TTC​CAG​C -3′); TNMD (F: 5′- GGA​CTG​GTG​TTT​GGT​ATC​CTG​G -3′, R: 5′- CTC​CAT​TGC​TGT​AGA​AAG​TGT​GC -3′); GAPDH (F: 5′- CCA​CTC​CTC​CAC​CTT​TGA​CG -3′, R: 5′- CCA​CCA​CCC​TGT​TGC​TGT​AG -3′)

#### 2.7.2 Western blot

To further evaluate tenogenic differentiation at the protein level, Western blot analysis was performed for SCX, TNC, and TNMD. *h*ADSCs were cultured under GM, DM, and DM supplemented with DHT extracts for 3 and 10 days. The cells were lysed using RIPA buffer containing protease inhibitors, and the total protein concentration was determined using a BCA protein assay kit (Thermo Fisher Scientific, USA). Equal amounts of protein were separated by SDS–PAGE and transferred onto polyvinylidene fluoride (PVDF) membranes using a wet transfer tank (Bio-Rad, USA). The membranes were blocked with 5% skim milk in Tris-buffered saline containing 0.1% Tween-20 (TBST) for 1 h at room temperature and were incubated overnight at 4 °C with primary antibodies against SCX, TNC, TNMD, and *β*-actin. After washing with TBST, the membranes were incubated with HRP-conjugated secondary antibodies for 1 h at room temperature. The protein bands were visualized using an enhanced chemiluminescence (ECL) reagent and imaged using a chemiluminescence detection system (Invitrogen, USA). *β*-Actin was used as an internal loading control.

#### 2.7.3 Immunocytochemistry

The expression of tenogenic markers was further evaluated by immunocytochemistry (ICC). *h*ADSCs were seeded on poly-L-lysine coated glass coverslips (2.5 × 10^4^ cells/cm^2^) and cultured in GM, DM, or DM supplemented with DHT extracts (DHT) for 10 days. The cells were fixed with 4% paraformaldehyde for 10 min at room temperature and permeabilized with 0.1% Triton X-100 for 5 min at room temperature. After blocking with 5% normal goat serum (NGS) for 1 h at room temperature, the cells were incubated overnight at 4 °C with primary antibodies against SCX, TNC, and TNMD. Subsequently, the cells were incubated with fluorescence-conjugated secondary antibodies for 1 h at room temperature in the dark. The coverslips were then inverted onto glass slides with a drop of mounting solution containing DAPI (4′,6-diamidino-2-phenylindole, Thermo Fisher Scientific, USA) to visualize the nuclei. The stained cells were observed using a fluorescence microscope (Axio Imager M2, Zeiss, Germany).

### 2.8 Micropulverization for injectable DHT

DHT derived from tendon tissues was micropulverized under cryogenic conditions. The resulting powder was classified into particle sizes of less than 100 μm. The size-selected powder was suspended in sterile normal saline (JW Pharmaceutical, Korea) to prepare an injectable formulation with concentrations optimized between 1% and 15% (*w/v*). The final formulation was loaded into 1.5 mL pre-filled syringes (Schott, Germany) containing 1 mL each and was sterilized by gamma irradiation.

### 2.9 Optimization and characterization of injectable DHT

#### 2.9.1 Particle size optimization

The micropulverized DHT powders have been fractionated into four size ranges (10–100, 100–200, 200–300, 300–400 μm). The particle size distribution and the specific surface area were analyzed under dry conditions with a laser-based particle size analyzer (0.8–2000 μm, ISO 13320:2020). Each fraction was suspended in sterile saline at 8% (*w/v*), loaded into 1.5 mL pre-filled syringes (1 mL per syringe, Schott, Germany), sterilized by gamma irradiation, and tested for injection force using a universal testing machine (2.5 kN load cell, 29 G needle, 100 mm/min plunger speed, ISO 11040–4). Three replicates were performed per size group.

#### 2.9.2 Concentration optimization

The particle size-optimized DHT powders (<100 μm) were suspended in the sterile saline at 3%, 8%, and 13% (*w/v*). Injection forces were measured under the same conditions as above, and average (F_avg_) and maximum (F_max_) forces were calculated in triplicate to determine the optimal formulation concentration.

#### 2.9.3 Macroscopic extrusion behavior

Formulations at 3%, 8%, and 13% (*w/v*) were extruded without a needle onto a flat surface to assess the viscosity, the texture, the uniformity, and the flow pattern. Visual inspection was used to compare handling and delivery properties across the concentrations.

#### 2.9.4 Final formulation characterization

The optimized injectable DHT (10–100 μm, 8% *w/v*) was sterilized by gamma irradiation. The particle size distributions of the post-sterilized samples were reanalyzed, and the injection forces were measured as described above. All tests were performed in triplicate to assure reproducibility and injectability.

### 2.10 *In vivo* studies using injectable DHT

#### 2.10.1 Surgical procedure and animal models

All experimental protocols were approved by the Institutional Animal Care and Use Committee (IACUC) of K-MEDI hub, a preclinical testing facility (Daegu, Korea) (Approval number: KMEDI-24060401-00) and the IACUC of D&T CRO Co., Ltd. (Approval number: 26E018). All procedures adhered to the institutional ethical standards for animal research and followed the Animal Research: Reporting of *In Vivo* Experiments (ARRIVE) guidelines. Male Sprague Dawley rats (8–9 weeks) were randomly assigned into four groups: (i) Native (consisting of intact rats without collagenase-induced injury or surgical intervention), (ii) control (consisting of collagenase-injured rats treated with PBS), (iii) injectable DHT, (iv) porcine collagen (*p*Collagen, clinically approved), (v) human acellular dermal matrix (*h*ADM, clinically approved). The animals were first anesthetized via intraperitoneal injection of Zoletil (30 mg/kg) combined with Xylazine (Rompun, 10 mg/kg). After anesthesia, the Achilles tendon region was disinfected with alcohol and povidone-iodine, and a 1 cm incision was made to expose the tendon. Collagenase I (20 µL) was applied to induce tendon injury (Supplementary Figure S4), and the incision site was sutured thereafter. One week after injury induction, 100 µL of each test material was injected into the injured tendon under the same anesthesia conditions described above. Evaluations were performed at 4 and 8 weeks post-implantation. At each endpoint, animals were euthanized with compressed CO_2_ using a gradual-fill method (30%–70% chamber volume/min) in accordance with IACUC guidelines.

#### 2.10.2 Collagen presence and inflammatory response at 1 week post-implantation

To evaluate inflammatory response and collagen deposition in the injured tendon, IHC staining was performed. The right hind limbs of the experimental animals were harvested at the designated endpoints, and tendon tissues were fixed, embedded in paraffin, and sectioned to prepare histological slides. The sections were subjected to IHC staining using primary antibodies against CD68 (ab955, Abcam, United Kingdom) and type I human collagen (ab138492, Abcam). After incubation with the appropriate secondary antibodies and chromogenic detection, the stained sections were observed under a light microscope. Quantitative analysis of the immunopositive areas was performed using ImageJ software (National Institutes of Health, USA). The positively stained regions within the tendon tissue were measured and expressed as a percentage of the total tissue area.

#### 2.10.3 MRI assessment

The structural changes and the healing progression were assessed using a 9.4 T small animal MRI scanner (Bruker BioSpec 94/20USR, Germany) with an RF RES 400 1H 112/086 QSN TO AD coil. The animals were anesthetized and positioned prone with hind limbs extended. High-resolution T2-weighted images were acquired using the RARE sequence. All MRI scans were performed at a consistent anatomical level centered on the collagenase-induced lesion site, ensuring that the same imaging depth was applied across all experimental groups. The hyper-intense regions indicating edema or inflammation were identified, and quantitative analysis was performed using ImageJ software (NIH, USA). For reproducible quantification, the tendon cross-sectional area was first manually delineated as a region of interest (ROI) on each slice by tracing the tendon boundary based on anatomical landmarks and signal contrast. ROI selection was consistently performed across groups using the same lesion-centered slices. Hyper-intense areas were then defined by applying an identical intensity threshold across all samples, set relative to the mean signal intensity of adjacent native-like tendon tissue. The high-signal area was normalized to the total tendon cross-sectional area and were averaged over three consecutive slices.

#### 2.10.4 Histological analysis

At the study endpoints, the animals were sacrificed, and the tissue samples were collected, fixed, paraffin-embedded, and sectioned. Collagen and GAG distribution were evaluated, and Picrosirius red staining was performed to assess the collagen arrangement and maturity. The stained sections were observed under a polarized light microscope (NP900, Nexcope, Korea).

#### 2.10.5 Bonar score evaluation

The histological sections were evaluated using the Bonar scoring system (Table 1), a semi-quantitative method commonly applied in tendon pathology to assess the cellularity, the collagen organization, the vascularity, and the ground substance changes. (Zabrzynska et al., 2021; Zabrzyński et al., 2021; Jaworski et al., 2022). Each parameter was graded from 0 to 3, with higher scores indicating more severe tissue degeneration. The total Bonar score was calculated by summing individual parameter scores to provide an overall assessment of tendon health and repair quality. Score interpretation was as follows: 0–3, normal or healthy tissue; 4–6, mild degeneration or minor abnormalities; 7–9, moderate degeneration with structural changes; 10–12, severe degeneration with significant cellular and structural alterations.

**TABLE 1:** Bonar histological semi-quantitative scoring system.

Parameter	Score 0	Score 1	Score 2	Score 3
Cell morphology	Normal, spindle-shaped cells	Some rounded cells, mild changes	Mostly rounded cells	Rounded or chondroid-like cells
Cellularity	Normal cell density	Slightly increased cells	Moderately increased cells	Markedly increased cells
Vascularity	No vascular proliferation	Slight vascular proliferation	Moderate vascular proliferation	Marked vascular proliferation
Ground substance	Normal ground substance	Slight increase	Moderate increase	Marked increase
Collagen fiber arrangement	Highly organized collagen fibers	Slightly disorganized collagen fibers	Moderately disorganized collagen fibers	Severely disorganized collagen fibers

#### 2.10.6 Functional assessment

Functional recovery of the Achilles tendon was evaluated using the AFI based on footprint analysis. Behavioral assessments were performed in Cohort 2 before induction of tendon injury, before administration of the test materials, and at 1, 4, and 8 weeks after implantation. For footprint analysis, the hind paws of the rats were coated with ink, and the animals were allowed to walk along a paper-lined corridor. Food pellets were placed at the end of the corridor to encourage continuous walking. After locomotion, the papers containing the footprints were collected and analyzed. From the footprints obtained after walking, the following parameters were measured: print length (PL), defined as the distance from the heel to the third toe; toe spread (TS), defined as the distance between the first and fifth toes; and intermediate toe spread (IT), defined as the distance between the second and fourth toes. Based on these parameters, the AFI was calculated according to the established formula. An AFI value close to 0 indicates normal gait function, whereas values between −20 and −40 represent mild impairment, −40 to −80 indicate moderate dysfunction, and values approaching −100 correspond to severe functional impairment.

$$\text{AFI}=74\times \frac{\left({PL}_{E}-{PL}_{N}\right)}{{PL}_{N}}+161\times \frac{\left({TS}_{E}-{TS}_{N}\right)}{{TS}_{N}}+48\times \frac{\left({IT}_{E}-{IT}_{N}\right)}{{IT}_{N}}-5$$

- E: Experimental (injured limb)
- N: Normal limb

#### 2.10.7 Biomechanical assessment

The biomechanical properties of the regenerated Achilles tendon were evaluated using a tensile testing system. At 8 weeks after administration of the test materials, the animals were euthanized using compressed CO_2_. The right hind limbs were harvested, and the Achilles tendons were carefully dissected. The excised tissues were wrapped in sterile gauze moistened with phosphate-buffered saline (PBS), sealed in plastic bags, and stored at −70 °C until analysis. Prior to mechanical testing, the frozen tendon samples were thawed at room temperature. Ultimate tensile strength and stiffness were measured using a universal testing machine (Instron, USA). Both ends of the tissue were placed between two layers of sandpaper, secured with cyanoacrylate adhesive, and mounted onto the testing device. Prior to tensile testing, all samples underwent identical preconditioning consisting of five cycles of loading between 0.05 N and 0.2 N. Subsequently, tensile testing was performed at a rate of 0.8 mm/s until failure. From the load–displacement curves obtained during testing, biomechanical parameters including stiffness and ultimate tensile strength were determined.

### 2.11 Statistical analysis

Data were expressed as mean ± standard deviation. Statistical analysis was performed using the GraphPad Prism software (Boston, MA, USA). All experiments were conducted with at least three independent replicates. Statistical differences were analyzed using the Student’s t-test for comparisons between two groups, and one-way analysis of variance (ANOVA) with Bonferroni post-hoc test for comparisons of more than two groups. A *p*-value of less than 0.05 was considered statistically significant.

## 3 Results and discussion

### 3.1 Optimization of decellularization and delipidation protocols for DHT preparation

**FIGURE 1:**
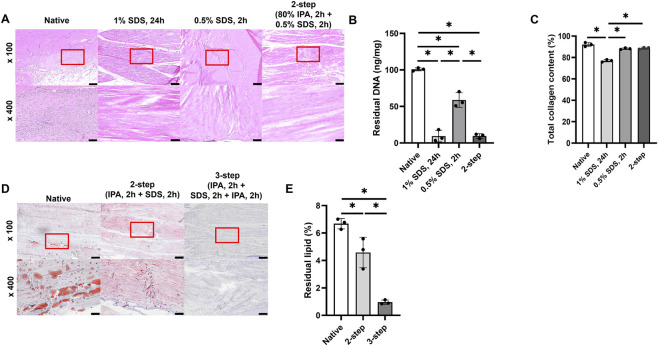
Optimization of decellularization and delipidation protocols for DHT preparation. **(A)** Representative H&E staining images of the native tendon and tendons treated with 1% SDS (24 h), 0.5% SDS (2 h), and the 2-step protocol (80% IPA for 2 h followed by 0.5% SDS for 2 h). Magnification: ×100 (top row) and ×400 (bottom row). Scale bars: 100 µm (top row) and 25 µm (bottom row). **(B)** Residual DNA content in native and treated tendons measured by biochemical assay. **(C)** Total collagen content in native and treated tendons measured by hydroxyproline assay. **(D)** Representative Oil Red O staining images of native tendon and tendons treated with the 2-step protocol and the 3-step protocol (80% IPA for 2 h, followed by 0.5% SDS for 2 h, followed by an additional 80% IPA treatment for 2 h). Magnification: ×100 (top row) and ×400 (bottom row). Scale bars: 100 µm (top row) and 25 µm (bottom row). **(E)** Residual lipid content in native tendon and tendons treated with the 2-step and the 3-step protocols. Data are presented as mean ± SD (*n* = 3). *p < 0.05.

We evaluated decellularization efficiency using H&E staining and residual DNA quantification (Figures 1A,B). Native tendons contained abundant nuclei, whereas both 1% SDS and IPA +0.5% SDS treatments effectively removed cellular components and significantly reduced residual DNA. In contrast, the 0.5% SDS group showed only partial decellularization. Collagen preservation was further assessed (Figure 1C). The 1% SDS group exhibited significantly reduced collagen content, whereas both IPA +0.5% SDS and 0.5% SDS treatments preserved collagen levels comparable to those of native tissues. Delipidation efficiency was evaluated by Oil Red O staining and residual lipid quantification (Figures 1D,E). While the 2-step treatment moderately reduced residual lipids, the 3-step treatment achieved near-complete lipid removal. These results demonstrate that the IPA-assisted protocol effectively removed cellular and lipid components under relatively mild processing conditions while preserving collagen integrity compared with conventional SDS-based decellularization methods. (Cartmell and Dunn, 2004).

Consistent with established decellularization principles, aggressive detergent- or solvent-based approaches, while effective, are often associated with an increased risk of ECM disruption and loss of bioactive components (Crapo et al., 2011). Rather than relying on prolonged detergent exposure, the present approach combines moderate SDS treatment with IPA-assisted delipidation to facilitate cellular membrane disruption and efficient removal of lipid-rich debris. IPA is known to solubilize lipid components and promote membrane destabilization, which can enhance subsequent detergent penetration and cellular residue clearance. Through this complementary mechanism, effective decellularization can be achieved while minimizing excessive chemical stress on the ECM. To further address potential donor heterogeneity, total collagen content was evaluated across tendon tissues obtained from donors of different sexes and age ranges, and no significant inter-donor differences were observed (Supplementary Figure S1). Donor demographic information is summarized in Supplementary Table S1. Such a balance between effective decellularization, enhanced delipidation, and matrix preservation is critical for the development of tendon-derived ECM biomaterials. Notably, IPA-assisted delipidation has been reported to preserve ECM integrity while reducing lipid-associated inflammatory responses. (Ventura et al., 2019; Nicholls et al., 2022). By reducing lipid-associated residues while maintaining ECM architecture, this strategy helps preserve the structural and biochemical cues required for tendon-specific regenerative scaffolds. Moreover, the retention of the structural proteins and the bioactive factors supports the development of injectable, tendon-specific ECM scaffolds with both mechanical integrity and biological functionality. Collectively, these findings suggest that IPA-assisted processing provides a mechanistically rational and translationally relevant strategy for generating human tendon–derived ECM biomaterials.

### 3.2 Characterization of DHT

**FIGURE 2:**
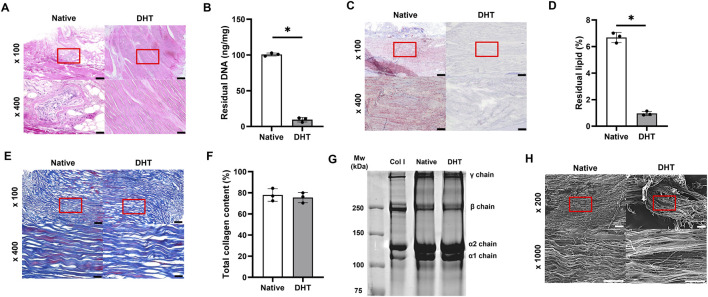
Characterization of DHT. **(A)** Representative H&E staining images of the native tendon and DHT. Magnification: ×100 (top row) and ×400 (bottom row). Scale bars: 100 µm (top row) and 25 µm (bottom row). **(B)** Residual DNA content in native tendon and DHT measured by biochemical assay. **(C)** Representative Oil Red O staining images of the native tendon and DHT. Magnification: ×100 (top row) and ×400 (bottom row). Scale bars: 100 µm (top row) and 25 µm (bottom row). **(D)** Residual lipid content in native tendon and DHT. **(E)** Representative MT staining images of the native tendon and DHT. Magnification: ×100 (top row) and ×400 (bottom row). Scale bars: 100 µm (top row) and 25 µm (bottom row). **(F)** Quantification of total collagen content in native tendon and DHT measured by hydroxyproline assay. **(G)** SDS-PAGE analysis of collagen type I (Col I) in native tendon and DHT. **(H)** Representative SEM images of the native tendon and DHT. Magnification: ×100 (top row) and ×400 (bottom row). Scale bars: 100 µm (top row) and 50 µm (bottom row). Data are presented as mean ± SD (*n* = 3). *p < 0.05.

We first evaluated the decellularization efficiency using H&E staining and residual DNA quantification (Figures 2A,B). Native tendon contained abundant nuclei, whereas DHT exhibited no detectable cellular structures and markedly reduced residual DNA. We next assessed lipid removal using Oil Red O staining and quantification (Figures 2C,D). Lipid droplets were observed in the native tissue but were largely absent in DHT, confirming effective delipidation. We then examined ECM preservation. MT staining demonstrated similar collagen distribution between the native tendon and DHT (Figure 2E), and hydroxyproline analysis further confirmed comparable collagen contents (Figure 2F). SDS-PAGE showed intact α1, α2, β-, and γ-collagen bands in DHT, consistent with the native tendon and type I collagen standards (Figure 2G). SEM imaging further revealed well-aligned and densely packed collagen fibrils in both groups (Figure 2H).

These findings confirm that the IPA-assisted protocol preserves key ECM features, including type I collagen and fibrillar architecture. Preservation of these structural characteristics is important because the aligned collagen hierarchy of tendon ECM plays a central role in maintaining tendon-specific mechanical properties and cell–matrix signaling (Kannus, 2000; Wang, 2006). This suggests that the processing conditions effectively removed cellular and lipid components while minimizing excessive chemical damage to the ECM (Crapo et al., 2011). Importantly, to address potential donor heterogeneity, total collagen content was additionally quantified across tendon tissues obtained from donors of different sexes and age ranges (Supplementary Figure S1), and no significant inter-donor differences were observed. This supports the batch-level consistency of the tendon ECM source and the reproducibility of the resulting DHT material (Gilbert et al., 2006).

### 3.3 Growth factor analysis of DHT

**FIGURE 3:**
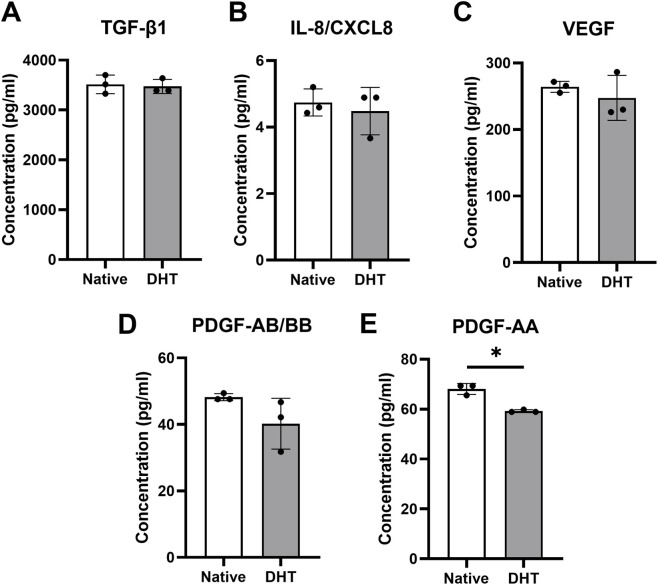
Growth factor analysis of DHT. Luminex assay was performed to compare growth factor levels in native tendon and DHT. Five key growth factors involved in tendon healing were analyzed **(A)** TGF-β1 **(B)** IL-8/CXCL8 **(C)** VEGF **(D)** PDGF-AA/BB **(E)** PDGF-AA. Data are presented as mean ± SD (*n* = 3). *p < 0.05.

We quantified the key growth factors to assess retention after decellularization (*n* = 3). TGF-*β*1 was well preserved in DHT compared with the native tendon (Figure 3A). IL-8/CXCL8 showed minimal change after decellularization (Figure 3B). VEGF and PDGF-AB/BB were modestly retained in DHT relative to the native tendon (Figures 3C,D), whereas PDGF-AA showed a slight but significant reduction after decellularization (*p < 0.05) (Figure 3E). Overall, most key bioactive molecules at the biologically relevant levels were found in DHT after decellularization. These factors, including TGF-*β*1, VEGF, IL-8, and PDGFs, are known to regulate inflammation, angiogenesis, and tendon remodeling, suggesting that DHT may provide biochemical cues supportive of tendon repair. (Rothrauff et al., 2017; Kimmerling et al., 2019; Liu et al., 2021). Importantly, these retained growth factor levels fall within physiologically relevant ranges reported for native tendon healing, where bioactivity is primarily mediated through ECM-bound presentation rather than soluble concentration. Thus, the preserved growth factor profile indicates that DHT maintains a native-like biochemical microenvironment rather than functioning as a supraphysiological growth factor delivery system.

### 3.4 Quantification of residual antibiotics, IPA and SDS in DHT

The residual antibiotics and chemicals in DHT were quantified to confirm the safety. High-performance liquid chromatography (HPLC) detected clear peaks at the retention times for antibiotic standards (Amphotericin B: 4.774 min; Penicillin G: 4.582 min; Streptomycin: 2.630 min), but no peaks were observed in DHT (Supplementary Figure S2A). Gas chromatography–mass spectrometry (GC-MS) analysis for IPA showed a distinct standard peak at 6.5–7.0 min, whereas the DHT samples exhibited none, indicating that the residual IPA levels below the detection limit (1 ppm) (Supplementary Figure S2B). Similarly, HPLC analysis for SDS revealed a standard peak at 3.0–3.5 min, absent in DHT, confirming the residual detergent below the detection limit (3 ppm) (Supplementary Figure S2C).

These results clarify that the subsequent washing steps in the DHT manufacturing process effectively removed residual antibiotics, IPA, and SDS. The absence of detectable chemical residues indicates that the processing workflow provides effective chemical clearance while maintaining material safety. Minimizing residual cytotoxic agents is particularly important for ECM-derived biomaterials, as residual solvents or detergents may otherwise trigger inflammatory responses or interfere with cellular behavior after implantation. (Crapo et al., 2011; Dziki et al., 2017). Therefore, the confirmed removal of these processing reagents supports the biocompatibility and translational safety of the injectable DHT platform for tendon repair applications.

### 3.5 *In vitro* assessment of cell response by DHT

**FIGURE 4:**
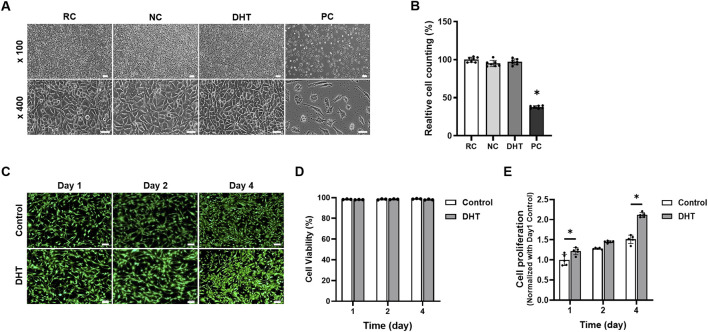
*In vitro* assessment of the cellular responses to DHT. **(A)** Representative images of L929 fibroblasts after 48 h incubation with extracts from the control and DHT groups. Magnification: ×100 (top row) and ×400 (bottom row). Scale bars: 100 µm (top row) and 25 µm (bottom row). **(B)** Relative cell counting in the reagent control (RC), negative control (NC), DHT and positive control (PC) groups (*n* = 7). *p < 0.05 (compared with all other groups). **(C)** Representative Live/Dead staining images of human tenocytes cultured with control or DHT extracts for 1, 2, and 4 days. Green and red fluorescence indicate live and dead cells, respectively. Magnification: ×100. Scale bar: 100 µm. **(D)** Cell viability in the control and DHT groups (*n* = 3). **(E)** Cell proliferation in the control and DHT groups on 1, 2, and 4 days measured by CCK-8 assay (*n* = 5). *p < 0.05.

First, the cytotoxicity of DHT was evaluated *in vitro*. The cell morphologies in the DHT-treated groups were comparable to those of the reagent control (RC) and negative control (NC) groups, with no rounding or detachment. Relative cell viability remained at 97%, corresponding to grade 1 (>80% viable cells), with no significant differences among the RC, NC, and DHT groups (Figures 4A,B). All three groups exhibited significantly higher viability compared with the positive control (*p < 0.05). We then assessed the tenocyte viability and proliferation using human tenocytes. Fluorescence imaging and quantitative intensity analysis indicated comparable viability in the DHT-treated and control groups on days 1, 2, and 4 (Figures 4C,D). CCK-8 assays showed time-dependent proliferation, with significant increase at days 1 and 4 in both groups (*p < 0.05) (Figure 4E).

These findings confirm the cytocompatibility of DHT and indicate that the decellularization and delipidation processes did not introduce detectable cytotoxic residues. Notably, these biological responses were evaluated using an extract-based approach in accordance with ISO 10993 guidelines, enabling assessment of bioactive factors released under physiologically relevant conditions while minimizing ECM denaturation. This approach is particularly relevant for tendon ECM, as prior studies have shown that aggressive solubilization methods can deplete non-collagenous proteins critical for tenogenic bioactivity. (Wong et al., 2016; Huang et al., 2024). Furthermore, the extract-based design of this study was intended to evaluate the bioactivity of DHT-derived soluble factors rather than direct cell–material interactions. However, direct cell–material interaction assays may provide additional insight into cellular responses to the preserved ECM architecture and should be further investigated in future studies. Together, these observations suggest that injectable DHT provides a biologically safe microenvironment capable of supporting tendon cell survival and early cellular activity, which are essential prerequisites for subsequent tissue regeneration.

### 3.6 Tenogenic differentiation of *h*ADSCs induced by DHT

#### 3.6.1 qRT*-PCR*

**FIGURE 5:**
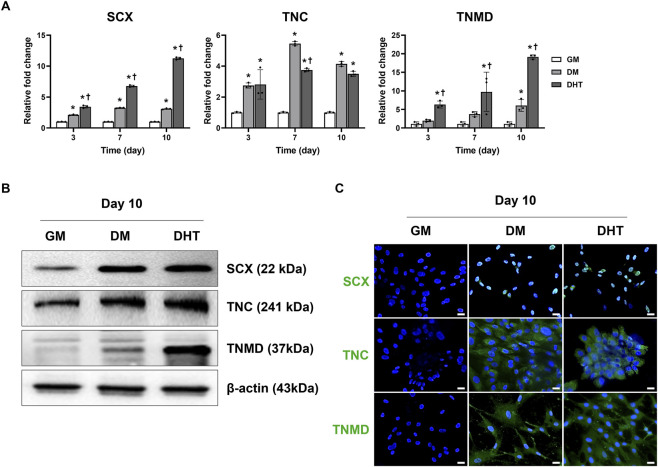
Tenogenic differentiation of *h*ADSCs induced by DHT. **(A)** qRT-PCR analysis of SCX, TNC and TNMD gene expression in *h*ADSCs cultured for 3, 7, and 10 days in control growth medium (GM), differentiation medium (DM), and DHT groups (*n* = 3). *p < 0.05 (compared with the control group at the same time point). *†p* < 0.05 (compared with DM group at the same time point). **(B)** Western blot analysis of SCX, TNC, and TNMD protein expression in *h*ADSCs cultured in GM, DM, and DHT groups for 10 days. β-actin was used as the loading control. **(C)** Immunocytochemistry (ICC) staining of SCX, TNC, and TNMD in *h*ADSCs cultured in GM, DM, and DHT groups for 10 days. Nuclei were counterstained with DAPI. Representative merged images are shown. Magnification: ×400. Scale bar: 25 µm.

We evaluated the tenogenic differentiation of human adipose-derived stem cells by qRT-PCR analysis of scleraxis (SCX), tenascin C (TNC), and tenomodulin (TNMD) mRNA expression at days 3, 7, and 10 (Figure 5A). SCX and TNMD expression in the DHT group progressively increased over time and reached the highest levels at day 10, indicating enhanced tenogenic differentiation compared with the GM and DM groups. In contrast, TNC expression, a marker associated with early tendon development, peaked at day 7 in both the DM and DHT groups and subsequently decreased by day 10, although expression levels remained elevated relative to the GM group.

#### 3.6.2 Western blot

The protein expression of SCX, TNC, TNMD, and β-actin was evaluated at day 10 under GM, DM, and DHT conditions (Figure 5B). SCX and TNC expression were in the GM group but markedly increased in both the DM and DHT. In contrast, TNMD expression increased progressively from GM to DM and was highest in the DHT group. β-actin expression remained consistent across all groups, confirming equal protein loading.

#### 3.6.3 ICC

ICC staining at day 10 further confirmed the expression of tenogenic markers (Figure 5C). Consistent with the Western blot results, SCX and TNC signals were enhanced in the DM and DHT groups, while TNMD expression was most prominent in the DHT group.

The ability of DHT to promote tenogenic differentiation was further supported at both the gene and protein levels. The significant upregulation of tendon-specific markers, including SCX and TNMD, were consistently upregulated in qRT-PCR, Western blot, and ICC analyses. SCX is a key transcription factor involved in early tendon lineage commitment, whereas TNMD is associated with tendon maturation and extracellular matrix organization. The increased expression of these markers suggests that DHT not only supports cell survival but also actively promotes tendon-specific phenotypic commitment. The protein-level results further demonstrated that SCX and TNC were strongly expressed in both DM and DHT groups, while TNMD expression was most pronounced in the DHT group, indicating enhanced maturation under DHT implantation. In addition, the transient elevation of TNC expression is consistent with its established role in early tendon remodeling and matrix reorganization. Together, these findings indicate that DHT provides a biochemical microenvironment conducive to coordinated tenogenic progression, supporting both early differentiations signaling and later-stage tendon maturation. These results highlight the regenerative potential of DHT as a bioactive matrix capable of guiding stem cell differentiation toward the tendon lineage.

### 3.7 Optimization and characterization of injectable DHT

DHT powders were fractionated into particle size ranges of 10–100, 100–200, 200–300, and 300–400 μm and suspended them in saline (8% *w/v*) to evaluate injectability. Injection force testing demonstrated that the 10–100 μm particles required substantially lower injection force than the larger particle groups and remained below the generally acceptable threshold of 40 N, indicating suitability for fine-gauge needle injection (Supplementary Figure S3A). Accordingly, 10–100 μm particles were used for subsequent formulation studies. (Lee et al., 2021; Micheels et al., 2024).

Formulations at 3%, 8%, and 13% (*w*/*v*) exhibited concentration-dependent increases in injection force (Supplementary Figure S3B). Although all formulations remained below the generally acceptable injection threshold, the 13% formulation approached the upper limit. Suggesting potential difficulty during manual injection. Microscopic extrusion analysis further demonstrates excessive fluidity in the 3% formulation and irregular cracking in the 13% formulation, whereas the 8% formulation showed continuous and homogeneous extrusion without fragmentation (Supplementary Figure S3C). Therefore, the 8% formulation was selected as the optimal condition for injectability and handling.

Following gamma sterilization, the optimized formulation retained a narrow particle size distribution and injection force characteristics (Supplementary Figures S3D, E). Rheological analysis further demonstrated clear shear-thinning behavior, with viscosity decreasing as shear increased, supporting injectability under clinically relevant conditions (Supplementary Figure S3F; Table 2).

**TABLE 2:** Viscosity of the injectable DHT as a function of shear rate.

Shear Rate [1/s]	Viscosity [mPa?s]	
​	Average	Standard deviation
0.1	227,620	108,790
1	17,860	6,980
10	1,730	440
100	270	80

These results highlight successful development of particle size- and concentration-optimized injectable DHT formulation. Powders within the 10–100 μm range at 8% (*w/v*) concentration provided optimal injectability through fine-gauge needles, with stable rheological behavior and sterilization tolerance. Unlike many previously reported injectable ECM materials that require solubilization or enzymatic digestion before use (Seif-Naraghi et al., 2012; Kim et al., 2016; Jiang et al., 2021; Li et al., 2024), the DHT used in this study was applied directly in its powder form without complete dissolution. This approach enabled the preparation of a ready-to-use injectable formulation while preserving the native tendon ECM structure. The combination of fine particle size and appropriate concentration allowed stable suspension behavior and low-resistance injection, while maintaining the structural characteristics of the ECM. These findings demonstrate that DHT powders can be formulated as an injectable system without prior solubilization, providing a structurally preserved ECM-based formulation with favorable injectability. However, a formal dose–response and implantation volume optimization study was not performed in the current work. Therefore, additional studies investigating the effects of formulation dose and implantation volume on therapeutic efficacy and tissue response will be necessary to further optimize the clinical applicability of injectable DHT.

### 3.8 *In vivo* studies using injectable DHT

To evaluate the *in vivo* therapeutic potential of injectable DHT, a collagenase-induced Achilles tendon injury model was established in rats. Collagenase was injected into the tendon to generate localized matrix disruption and inflammatory degeneration, providing a reproducible platform for subsequent implantation and healing assessment (Supplementary Figure S4). The collagenase-induced Achilles tendinopathy model is widely used in preclinical tendon regeneration studies because it reliably induces key pathological features, including collagen disruption and inflammatory infiltration within 7 days. These characteristics enable consistent evaluation of therapeutic interventions targeting tendon degeneration. The selected implantation volume (100 µL) is also consistent with previous rat Achilles tendinopathy studies using injectable therapeutics to ensure sufficient local delivery within the tendon lesion site. However, it should be noted that collagenase-induced injury does not fully recapitulate the chronic mechanical overuse mechanisms underlying most clinical tendinopathy cases (Capkin et al., 2025). Therefore, further validation in chronic overuse or large-animal models will be important to strengthen translational relevance. Moreover, given that adult rats exhibit an accelerated aging timeline compared with humans, the 4–8 weeks observation period in this study may reflect a substantially longer progression window in human tendinopathy, allowing meaningful evaluation of tendon remodeling and healing responses (Sengupta, 2013). Importantly, beyond these model considerations, the injectable DHT demonstrated favorable *in vivo* handling characteristics, enabling localized delivery within the tendon defect without noticeable dispersion into surrounding tissues. Representative gross images obtained at the time of sacrifice further revealed that residual DHT materials remained localized within the treated Achilles tendon region at 8 weeks post-implantation, indicating sustained *in situ* retention of the injected ECM particles. Such localized delivery and prolonged retention may support continuous matrix–tissue interaction and provide structural and biochemical cues that facilitate tendon remodeling during the healing process.

### 3.9 Collagen presence and inflammatory response 1 week after implantation

**FIGURE 6:**
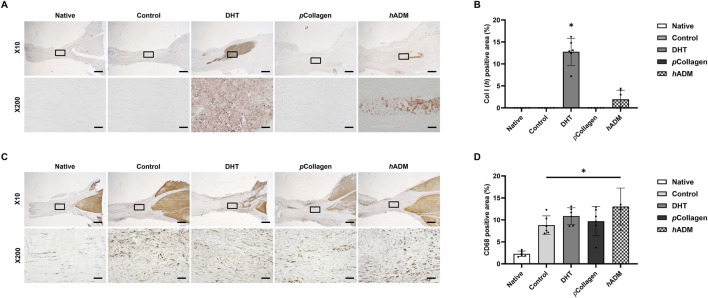
Collagen presence and inflammatory response at 1 week post-implantation. **(A)** Representative IHC staining images of human collagen type I (Col I (*h*)) in the Native, Control, DHT, *h*ADM, and *p*Collagen groups at 1 week post-implantation. Magnification: ×10 (top row) and ×200 (bottom row). Scale bars: 1,000 µm (top row) and 50 µm (bottom row). **(B)** Quantification of human collagen type I–positive area (%) from **(A)**. The positive area was calculated as the percentage of stained area relative to the total tendon area at the implantation site using images acquired at ×10 magnification (*n* = 6 per group). *p < 0.05 (compared with all other groups). **(C)** Representative IHC staining images of CD68 in the Native, Control, DHT, *h*ADM, and *p*Collagen groups at 1 week post-implantation to evaluate macrophage infiltration as an indicator of the inflammatory response. **(D)** Quantification of CD68-positive area (%) from **(C)**. The positive area was calculated as the percentage of stained area relative to the total tendon area at the implantation site using images acquired at ×10 magnification (*n* = 6 per group). *p < 0.05 (compared with the Native group).

To evaluate inflammatory response and collagen presence at the early stage of tendon healing, IHC staining for CD68 and type I human collagen was performed at 1 week post-implantation. Representative images are shown at low (×10) and high (×200) magnifications, where ×10 images provide an overview of the entire tendon tissue and ×200 images highlight the implantation site. Type I human collagen staining was conducted to assess the retention of implanted human-derived ECM materials. No detectable staining was observed in the Native, Control, or *p*Collagen groups, whereas the DHT-treated group exhibited the strongest positive staining, followed by the *h*ADM group (Figure 6A). These findings indicate that injectable DHT remained localized within the tendon region during the early stage after implantation. Quantitative analysis further confirmed that the DHT group exhibited the highest collagen-positive area among all groups, whereas only minimal staining was detected in the *h*ADM group (Figure 6B). CD68 staining was performed to evaluate macrophage infiltration as an indicator of inflammatory response. Minimal CD68 expression was observed in the Native group, whereas comparable levels of staining were detected across all injured groups (Figure 6C), indicating increased macrophage infiltration following tendon injury. Quantitative analysis further confirmed that all experimental groups exhibited significantly higher CD68-positive areas than the Native group, with no substantial differences observed among the treated groups (Figure 6D).

The observed retention of human collagen in DHT suggests that the particle-based injectable DHT enables effective localization of ECM components within the tendon defect without rapid dispersion (Singelyn et al., 2009; Seif-Naraghi et al., 2012). Compared to *h*ADM, the stronger collagen signal in G3 indicates a higher degree of material retention at the early stage after implantation. Such localized retention may facilitate sustained matrix–tissue interaction, which is important for guiding early-stage tendon remodeling (Badylak et al., 2011; Brown and Badylak, 2014). The low CD68 expression observed in the Native group reflects the absence of injury and represents the baseline physiological condition. In contrast, the increased CD68 expression observed across all experimental groups is primarily attributed to collagenase-induced tendon injury and the surgical intervention rather than the implanted materials themselves. Collagenase injection is known to induce matrix disruption and inflammatory cell infiltration, leading to elevated macrophage presence during the early phase of tendon healing (Perucca Orfei et al., 2016; Luo et al., 2023). While macrophage infiltration is a normal component of early tissue repair, excessive or prolonged inflammation may impair regenerative outcomes (Wynn and Vannella, 2016; Nicholls et al., 2022). In this context, the comparable levels of CD68 expression among the treated groups suggest that the implanted materials, including DHT, do not exacerbate the injury-induced inflammatory response. Rather, the retained ECM components in DHT may contribute to a localized microenvironment that supports tissue remodeling while interacting with the host inflammatory response. However, detailed macrophage phenotyping (e.g., CD163) and cytokine profiling were not included in the current study. Therefore, the immunomodulatory effects of DHT should be interpreted with caution, and comprehensive quantitative immune profiling will be required in future studies to further clarify the host immune response to injectable DHT.

### 3.10 MRI assessment

**FIGURE 7:**
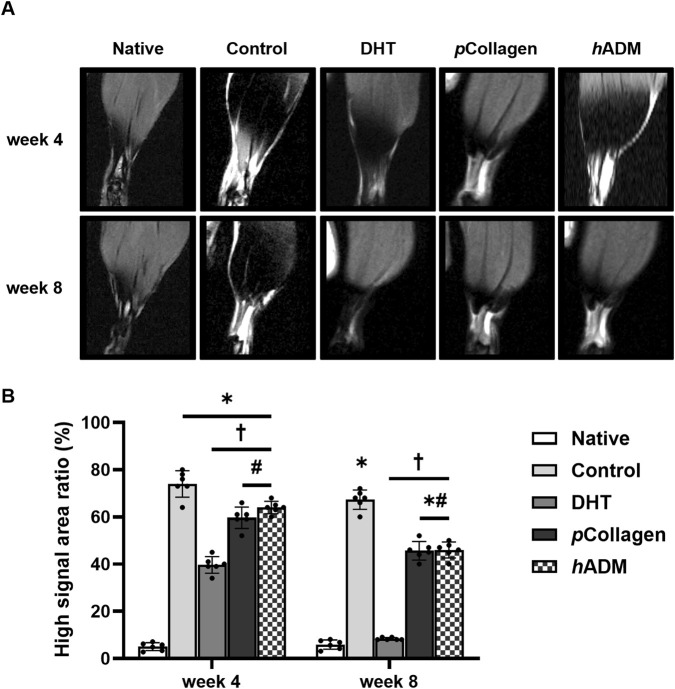
MRI assessment. **(A)** Representative MRI images of the implantation site at baseline (0 week), 4 weeks, and 8 weeks post-implantation. Baseline images were acquired 1 week after collagenase injection immediately prior to implantation. **(B)** Quantification of high-signal area ratio (%) in each group at 4 and 8 weeks post-implantation. The high-signal area ratio was calculated as the percentage of the tendon region exhibiting high signal intensity. Values were averaged from three consecutive MRI slices per animal, and each animal was treated as an independent biological replicate (*n* = 6 per group). *p < 0.05 (compared with the Native group at each time point). *†p* < 0.05 (compared with the Control group at each time point). *#p* < 0.05 (compared with the DHT group at each time point).

MRI was used to non-invasively monitor tissue healing in a collagenase-induced rat Achilles tendon injury model (Figure 7A; Supplementary Figure S5). T2-weighted RARE images acquired at 4 and 8 weeks revealed hyper-intense areas around the Achilles tendon, indicative of inflammation and edema (Figure 7B). At 4 weeks, all injured groups exhibited elevated signal intensity compared to the Native tendon, whereas all implantation groups showed reduced hyper-intense areas relative to the Control group. Among them, the DHT group demonstrated the lowest signal intensity, suggesting a more favorable early healing response. At 8 weeks, the DHT group exhibited near-native signal intensity, whereas the Control, *p*Collagen, and *h*ADM groups still showed elevated hyper-intense areas. Notably, the DHT group maintained the lowest signal intensity among all implantation groups, indicating more effective resolution of inflammation and improved tissue remodeling over time.

The MRI findings collectively demonstrate that injectable DHT effectively modulates the inflammatory response and promotes tissue recovery throughout the healing process (Sharma and Maffulli, 2005; Docheva et al., 2015). Elevated hyper-intense signals observed across all experimental groups relative to the Native tendon reflect injury-induced inflammation and edema following collagenase treatment (Perucca Orfei et al., 2016). In contrast, the consistently lower signal intensity observed in the DHT group compared to the Control and other implantation groups indicates a more favorable healing response. Interestingly, the early CD68 analysis at 1 week showed comparable macrophage infiltration across all treated groups, suggesting that DHT did not immediately suppress the initial inflammatory response induced by collagenase injury and surgical intervention. However, the MRI findings at 4 and 8 weeks demonstrated that the DHT group exhibited the most pronounced reduction in hyper-intense areas over time, indicating more efficient long-term resolution of inflammation compared to the other implantation groups. Importantly, the progressive reduction in hyper-intense areas observed in the DHT group suggests efficient resolution of inflammation and restoration of tissue integrity. The near-native signal intensity achieved in the DHT group further supports its ability to facilitate recovery toward a physiologically normal tendon state. In contrast, although *p*Collagen and *h*ADM groups showed partial improvements, their relatively higher signal intensities indicate less effective resolution of inflammation. These findings indicate that injectable DHT not only modulates early inflammatory responses but also supports sustained resolution of inflammation and tissue remodeling (Sadtler et al., 2016). The superior MRI outcomes observed in the DHT group may be attributed to the preserved native ECM architecture and bioactive components, which facilitate coordinated tissue remodeling and regulation of tissue hydration and edema within the healing tendon (Singelyn et al., 2009; Seif-Naraghi et al., 2012).

### 3.11 Histological analysis and bonar score evaluation

**FIGURE 8:**
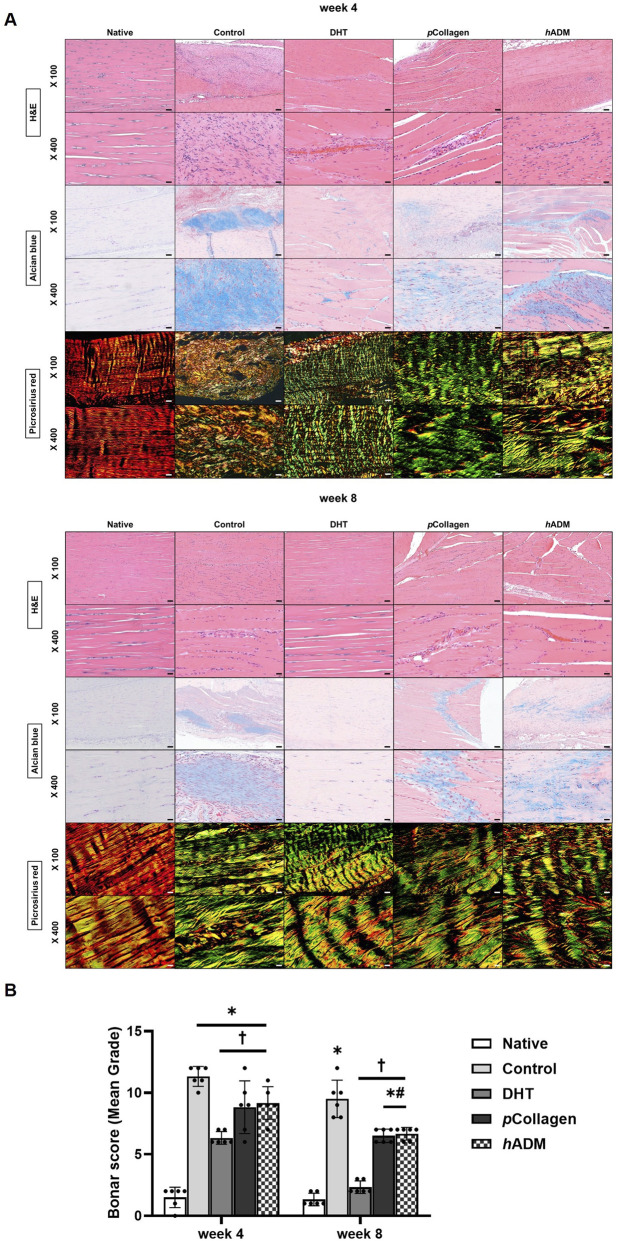
Histological analysis and Bonar score evaluation. **(A)** Representative H&E, Alcian blue, and Picrosirius red staining images of tendon tissues from the Native, Control, DHT, *h*ADM, and *p*Collagen groups at 4 and 8 weeks post-implantation. The first and second rows show H&E staining; the third and fourth rows show Alcian blue staining; and the fifth and sixth rows show Picrosirius red staining. Magnification: ×100 (odd-numbered row) and ×400 (even-numbered row). Scale bars: 100 µm in the first, third, and fifth rows and 25 µm in the second, fourth, and sixth rows. **(B)** Total Bonar scores based on histological analysis (*n* = 6 per group). Values were averaged from multiple tissue sections per animal, and each animal was treated as an independent biological replicate. *p < 0.05 (compared with the Native group at each time point). *†p* < 0.05 (compared with the Control group at each time point). *#p* < 0.05 (compared to the DHT group at time point).

We evaluated tendon healing in rat Achilles tendon tissues harvested at 4 and 8 weeks following implantation of PBS(Control), injectable DHT, *h*ADM, or *p*Collagen (*n* = 6) (Figure 8A). H&E staining revealed distinct differences in tissue morphology and architecture among the groups. DHT-treated tendons exhibited elongated, spindle-shaped tenocyte-like cells with aligned nuclei, indicative of restored tendon-like structure, whereas the Control group showed rounded cells and disrupted ECM. The *h*ADM and *p*Collagen groups demonstrated partial morphological recovery. Alcian blue staining further showed reduced glycosaminoglycan accumulation in the DHT group, particularly at 8 weeks, whereas the PBS group retained abundant positive regions. Semi-quantitative Bonar scoring was next performed to assess tendon healing quality based on cellularity, collagen organization, vascularity, and ground substance (Figure 8B; Supplementary Figure S6). At 4 weeks, the Control group exhibited the highest Bonar score, whereas all implantation groups showed improved histological outcomes, with the DHT group demonstrating the greatest degree of recovery. At 8 weeks, the DHT group exhibited near-native histological characteristics and maintained the lowest Bonar score, while the *p*Collagen and *h*ADM groups showed less complete structural recovery. Consistent with these findings, DHT-treated tendons exhibited reduced MRI-detected inflammation and edema, together with highly organized collagen fiber alignment under polarized picrosirius red staining. In contrast, collagen fibers in the PBS group remained disorganized, whereas the *p*Collagen and *h*ADM groups showed intermediate collagen maturation.

These observations indicate that tendon-specific ECM derived from DHT supports more organized collagen remodeling and improved histological recovery compared with non–tendon-specific ECM scaffolds (Thomopoulos et al., 2010; Gwon et al., 2023). The restoration of spindle-shaped cell morphology, reduced GAG deposition, and improved collagen alignment collectively suggest that DHT provides tendon-mimetic biochemical and structural cues that guide regenerative healing (Thomopoulos et al., 2010; Gwon et al., 2023). In particular, the highly aligned collagen architecture observed in the DHT group is indicative of more mature tendon remodeling, which is essential for restoring the anisotropic mechanical properties required for physiological load transfer. The reduced GAG accumulation further suggests attenuation of scar-like matrix deposition and progression toward a more tendon-like extracellular matrix composition during healing. Accordingly, tendon-derived ECM such as DHT may offer advantages over currently used scaffolds by promoting more complete structural recovery of injured tendon tissue (Glenski and Conner, 2009; Thomopoulos et al., 2010; Crapo et al., 2011; Sadtler et al., 2016; Zhang et al., 2022; Gwon et al., 2023). These findings also suggest that preservation of tissue-specific ECM ultrastructure and bioactive composition may play an important role in directing constructive tendon remodeling beyond simple defect filling or mechanical support.

### 3.12 Functional and biomechanical assessment

**FIGURE 9:**
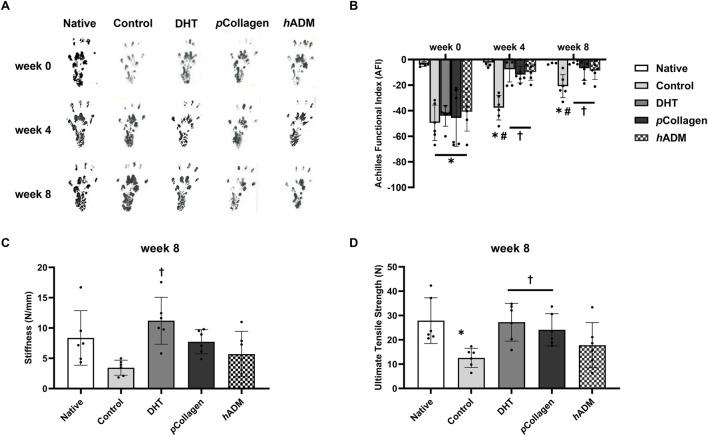
Functional and biomechanical assessment. **(A)** Representative images of the Achilles Functional Index (AFI) assessment using the footprint analysis method. Representative images of the experimental setup and recorded footprints are shown. Representative footprints from each group at weeks 0, 4, and 8 are presented. Print length (PL), toe spread (TS), and intermediary toe spread (IT) were measured to calculate AFI values. **(B)** Quantification of AFI values in each experimental group at weeks 0, 4, and 8 post-implantation based on footprint analysis (*n* = 6 per group). For each animal, AFI values were calculated using PL, TS, and IT parameters, and each animal was treated as an independent biological replicate. *p < 0.05 (compared with the Native group at each time point). *†p* < 0.05 (compared with the Control group at each time point). *#p* < 0.05 (compared with the DHT group at each time point). **(C)** Stiffness measured by uniaxial tensile testing at 8 weeks post-implantation (*n* = 6 per group). **(D)** Ultimate tensile strength measured by uniaxial tensile testing at 8 weeks post-implantation (*n* = 6 per group). Mechanical properties were compared among groups. *p < 0.05 (compared with the Native group). *†p* < 0.05 (compared with the Control group).

We evaluated functional recovery following tendon injury using the AFI at 0, 4, and 8 weeks post-implantation (*n* = 6). Representative footprint images and quantitative AFI analysis demonstrated progressive, time-dependent recovery in all implantation groups compared to the Control group (Figures 9A,B). At week 0, all injured groups showed severe functional impairment relative to the Native tendon. At 4 weeks, the DHT group exhibited the greatest functional improvement, whereas the Control group remained substantially impaired. By 8 weeks, the DHT group reached near-native AFI levels, while the *p*Collagen and *h*ADM groups showed partial recovery and the Control group continued to exhibit persistent functional deficits. Biomechanical evaluation at 8 weeks further supported these findings (Figures 9C,D). Tendon stiffness was significantly increased in the DHT group compared with the Control group, whereas no significant differences were observed among the other groups. The Control group exhibited reduced mechanical strength relative to the Native tendon, while the DHT and *p*Collagen groups showed significantly improved mechanical properties compared to the Control group. Among the implantation groups, the DHT group demonstrated the greatest biomechanical recovery, with values approaching those of the Native tendon.

The combined functional and biomechanical analyses demonstrate that injectable DHT effectively promotes tendon recovery across both locomotor and mechanical outcomes. The near-native AFI values in the DHT group indicate substantial restoration of gait function, whereas the Control group showed persistent impairment and the *p*Collagen and *h*ADM groups exhibited only partial recovery. Functional recovery is closely associated with the restoration of collagen organization, mechanical integrity, and cell–matrix interactions during tendon healing. (Sharma and Maffulli, 2005; Docheva et al., 2015). The superior functional outcomes in the DHT group are consistent with the histological and MRI findings, suggesting coordinated tissue remodeling that translates into improved biomechanical performance. Consistently, biomechanical testing showed that DHT restores the load-bearing capacity of injured tendons to near-native levels. Tendon mechanical competence depends on collagen organization and maturation, which determine ultimate tensile strength and stiffness (Screen et al., 2004; Wang, 2006). Interestingly, the increased stiffness observed in the DHT group may reflect enhanced collagen organization and ongoing matrix remodeling during tendon healing. Because remodeling may not be fully completed at 8 weeks, the elevated stiffness could also be associated with incomplete viscoelastic normalization during the maturation phase. Among the implantation groups, DHT and *p*Collagen exhibited significantly improved mechanical properties compared to the Control group, whereas the *h*ADM group showed only partial improvement without statistical significance. The preserved ECM architecture in DHT may further support these outcomes by providing structural and biochemical cues for matrix remodeling (Badylak et al., 2011; Sadtler et al., 2016). Collectively, these findings indicate that injectable DHT translates structural repair into both functional and mechanical recovery, supporting its potential for durable tendon regeneration.

## 4 Conclusion

In this study, we developed an injectable human tendon–derived extracellular matrix (DHT) using a mild, dual IPA-assisted decellularization and delipidation process. This approach effectively removed cellular and lipid components while preserving key ECM constituents, including type I collagen and bioactive factors. The resulting DHT supported tenocyte viability and promoted the tenogenic differentiation of human adipose-derived stem cells *in vitro*, as evidenced by the upregulation of SCX, TNMD, and temporally regulated TNC expression. *In vivo*, injectable DHT significantly enhanced tendon healing in a collagenase-induced rat Achilles tendon injury model. DHT implantation improved matrix architecture and reduced Bonar scores, indicating superior histological recovery. MRI analysis demonstrated effective resolution of inflammation, while functional assessment showed near-complete restoration of gait. Furthermore, biomechanical testing confirmed recovery of load-bearing capacity to near-native levels, indicating restoration of mechanical integrity. Collectively, these findings demonstrate that injectable DHT not only promotes structural and biological repair but also translates these effects into functional and mechanical recovery. The minimally invasive delivery and robust regenerative capacity of DHT highlight its strong translational potential as a tendon-specific therapeutic platform. Future studies in large-animal models and long-term evaluations will be essential to further validate its clinical applicability and durability.

## Data Availability

The raw data supporting the conclusions of this article will be made available by the authors, without undue reservation.
