# Supplementary material for: Injectable human tendon extracellular matrix via isopropanol-assisted delipidation and mild decellularization for tendon regeneration
Source: Front Bioeng Biotechnol. 2026 Jul 7;14:1745558. doi: 10.3389/fbioe.2026.1745558 (PMC13385181; doi:10.3389/fbioe.2026.1745558)
Supplement: Supplementary file 1 [file Supplementaryfile1.docx]

Supplementary Material

Injectable Human Tendon Extracellular Matrix via Isopropanol-Assisted Delipidation and Mild Decellularization for Tendon Regeneration

Jung Ki Lee^1^, Jiyeon Mun^1^, Hee Yeon Kim^1^, Yeon-Seop Jung^2^, Sang-Hyun An^2^, Changsoo Kim^1^, Kee-Won Lee^1^*

^1^R&D Center, L&C BIO Co., Ltd., 82, Naruteo-ro, Seocho-gu, Seoul, Republic of Korea

^2^Preclinical Research Center, Daegu-Gyeongbuk Medical Innovation Foundation (K-MEDI hub), 80, Cheombok-ro, Dong-gu, Daegu, Republic of Korea

*** Correspondence:**Kee-Won Lee, Ph.D.
[klee92@lncbio.co.kr](mailto:klee92@lncbio.co.kr)

**Table S1. Demographic information of human tendon donors used for ECM preparation.** The upper table presents individual donor information, and the lower table summarizes the donor cohort. Tendon tissues were obtained from seven donors (age range: 40–75 years; 5 males and 2 females).

| **Donor No.** | **Age (years)** | **Sex** |
| --- | --- | --- |
| Donor 1 | 40 | Male |
| Donor 2 | 42 | Male |
| Donor 3 | 64 | Male |
| Donor 4 | 69 | Female |
| Donor 5 | 71 | Male |
| Donor 6 | 72 | Male |
| Donor 7 | 75 | Female |

| **Parameter** | **Value** |
| --- | --- |
| **Number of donors** | 7 |
| **Sex** | 5 Male / 2 Female |
| **Age range** | 40–75 years |

**Figure S1.** **Donor variability analysis of total collagen content in human tendon tissues.**
Quantification of total collagen content in tendon tissues obtained from donors of different sexes and age ranges. Data are presented as mean ± SD (*n* = 2). No significant differences were observed among donors.


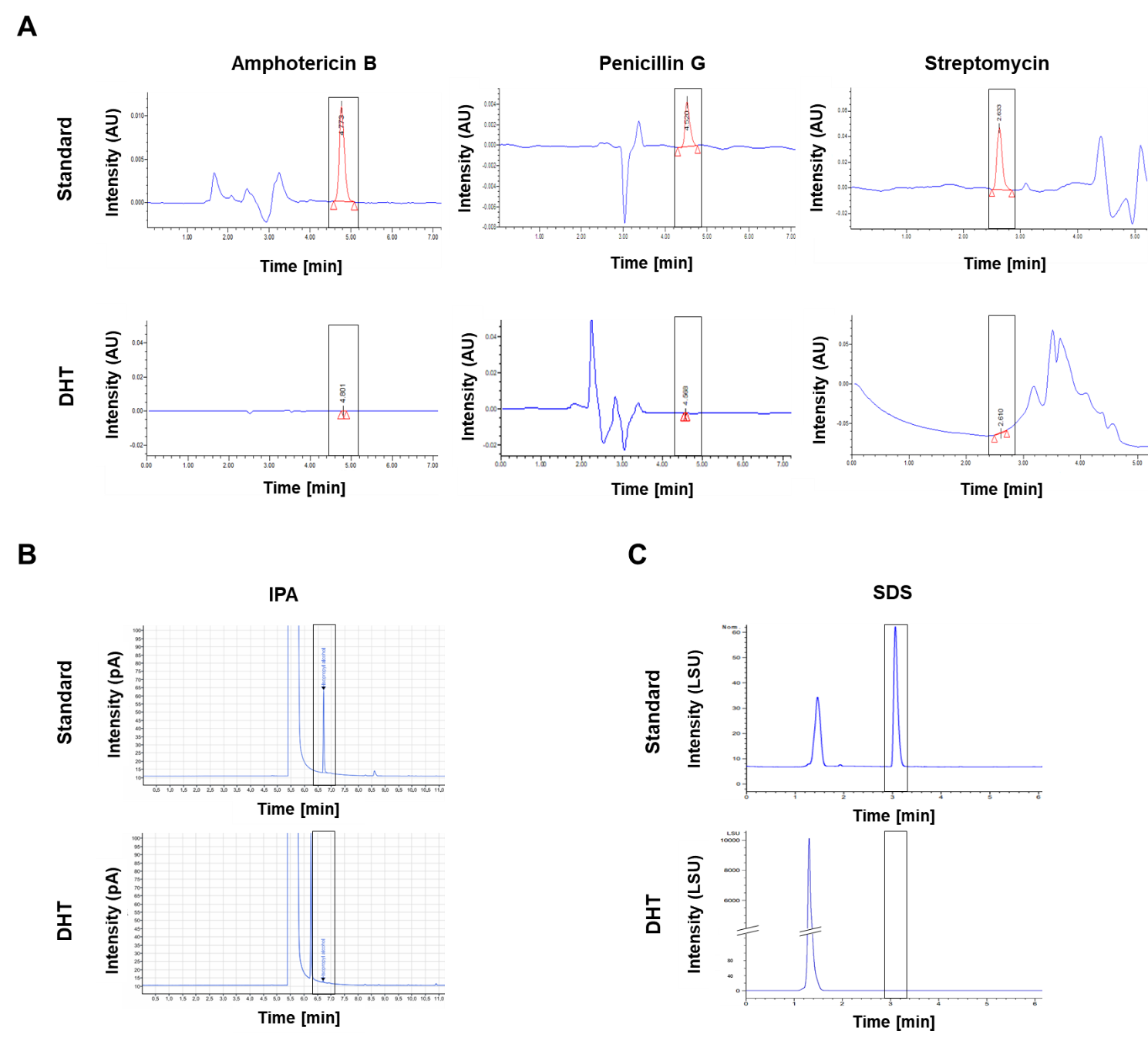
 **Figure S2.** **Quantification of residual antibiotics, IPA and SDS in DHT.** (A) Representative HPLC chromatograms of Amphotericin B, Penicillin G, and Streptomycin in DHT, with corresponding standards for comparison. (B) Representative GC-MS chromatograms of IPA in DHT, showing standard (top row) and DHT samples (bottom row). (C) Representative HPLC chromatograms of SDS in DHT, showing standard (top row) and DHT samples (bottom row).


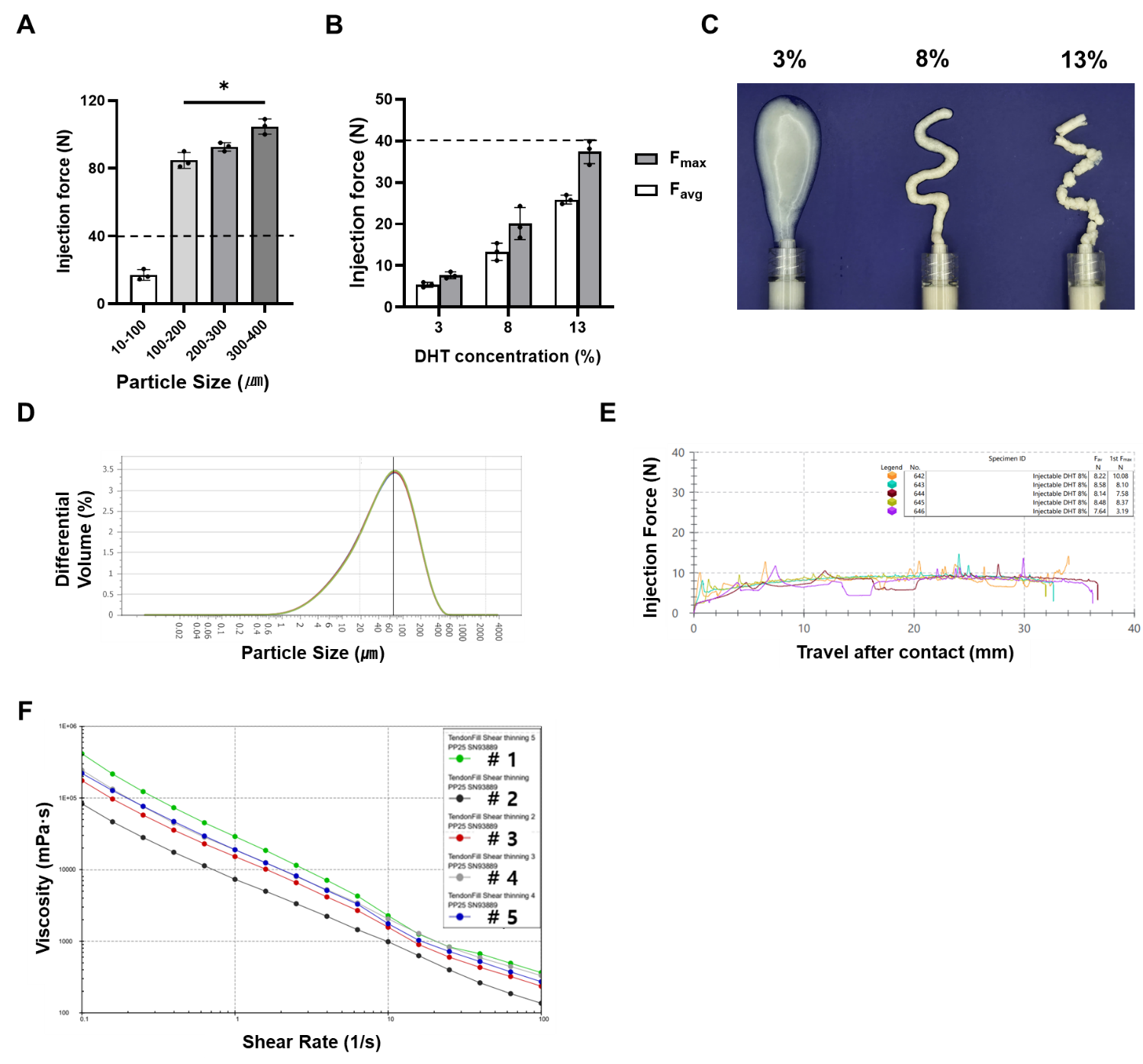


**Figure S3. Optimization and Characterization of injectable DHT**. (A) Injection forces were measured from samples with different particle size ranges using a uniaxial tensile testing machine equipped with a 29 G needle (*n* = 3). **p* < 0.05 compared with 10-100㎛ group. (B) Average (F_avg_) and maximum (F_max_) injection forces of injectable DHT formulations at concentrations of 3%, 8%, and 13% using a 29 G needle (*n* = 3). (C) Visual comparison of injectable DHT formulations at 3%, 8%, and 13% concentrations, showing differences in physical appearance and extrusion behavior. (D) Particle size distribution analysis of injectable DHT at different concentrations (*n* = 3). The vertical line indicates the modal particle diameter corresponding to the most frequently observed particle size in each sample. (E) Injectability test using a 29 G needle, showing force (N) versus travel distance (mm) curves for each sample (*n* = 5). Each curve is color-coded and labeled by sample ID, demonstrating consistent injectability with low resistance. (F) Shear-thinning rheological behavior of injectable DHT, showing viscosity (mPa·s) as a function of shear rate (1/s) (*n* = 5). The progressive decrease in viscosity with increasing shear rate demonstrates pronounced shear-thinning behavior relevant to injectability and handling properties.


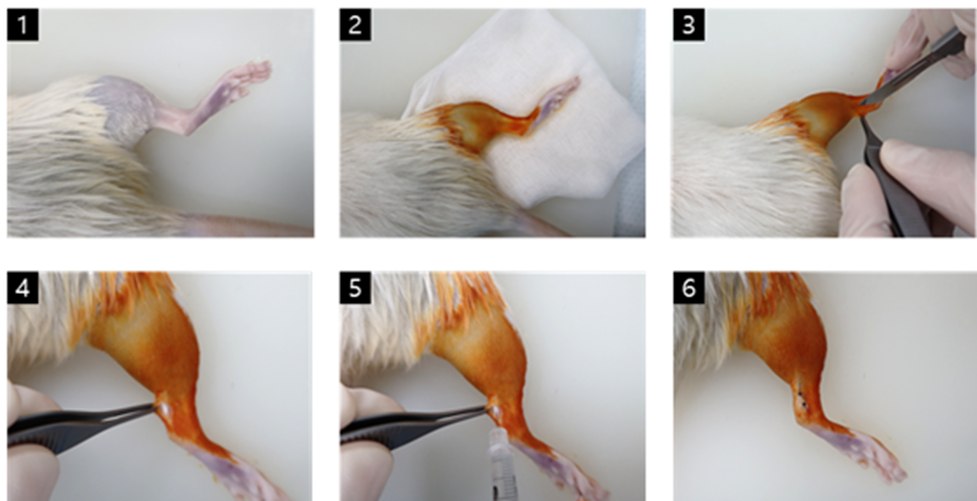


**Figure S4.** **Surgical procedure for the *in vivo* Achilles tendon injury model and injectable DHT administration.** Photographs show (1) shaving of the skin around the Achilles tendon, (2) disinfection of the surgical area with iodine solution, (3) exposure of the Achilles tendon, (4) fixation of the tendon, (5) collagenase injection into the tendon, and (6) closure of the incision site by suturing.


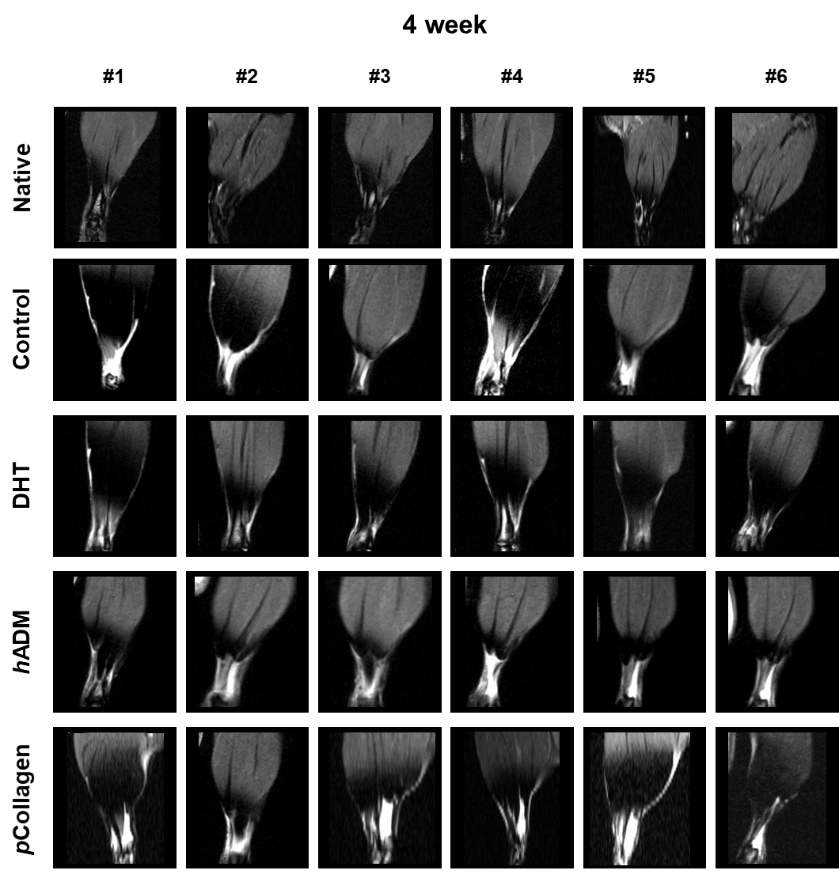


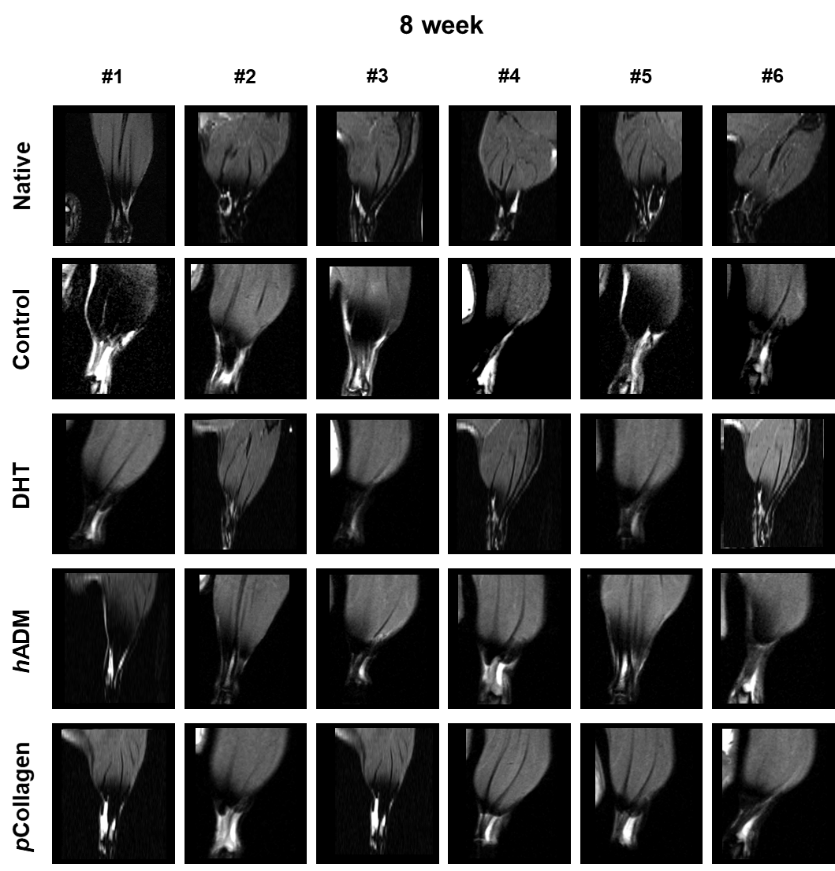


**Figure S5. Representative MRI images and corresponding signal intensity maps.** Representative MRI images of rat Achilles tendons at 4 and 8 weeks post-implantation in the Native, Control, DHT, *h*ADM, and *p*Collagen groups (*n* = 6 per group). Raw MRI data for all samples are provided.


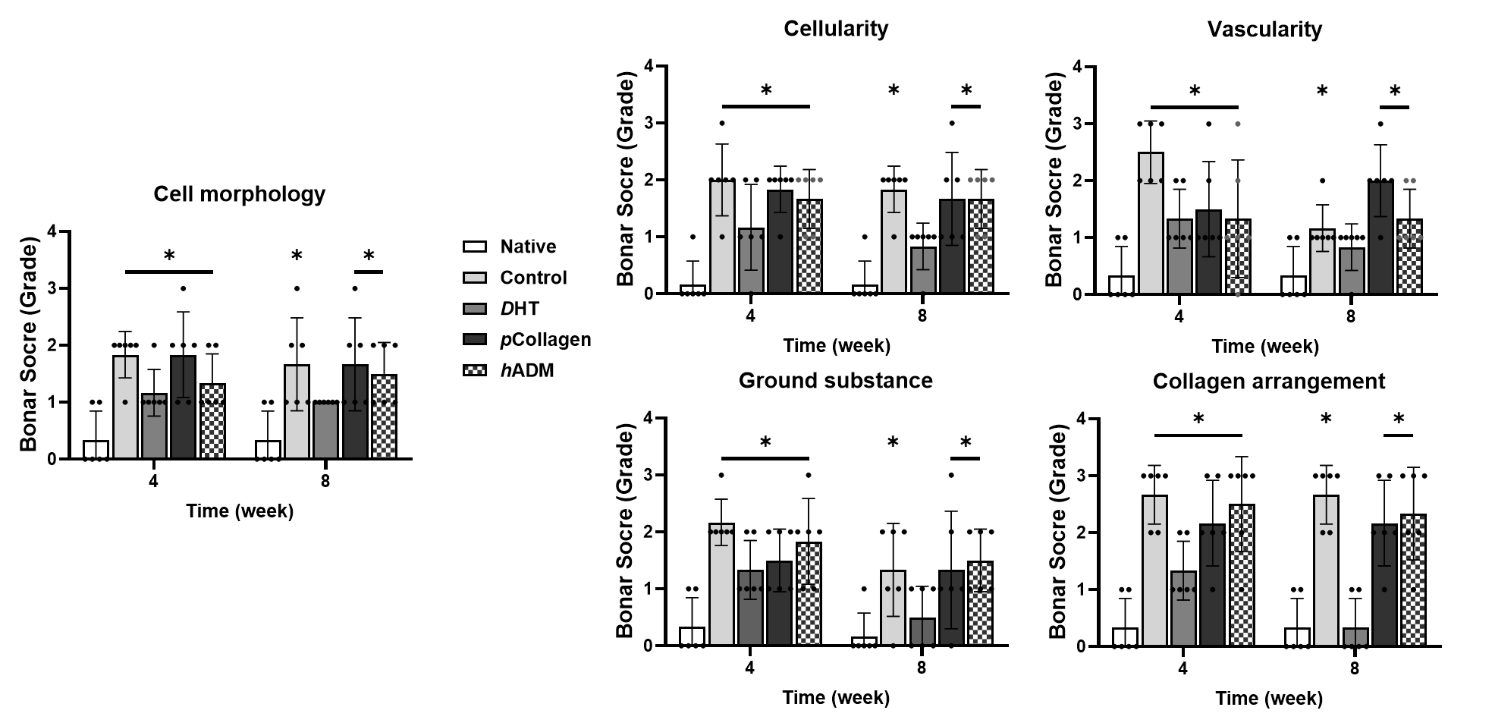


**Figure S6. Raw data for Bonar score analysis.** Quantitative Bonar score data of regenerated tendon tissues for each experimental group (*n* = 6 per group). **p* < 0.05 (compared with the Native group at each time point).


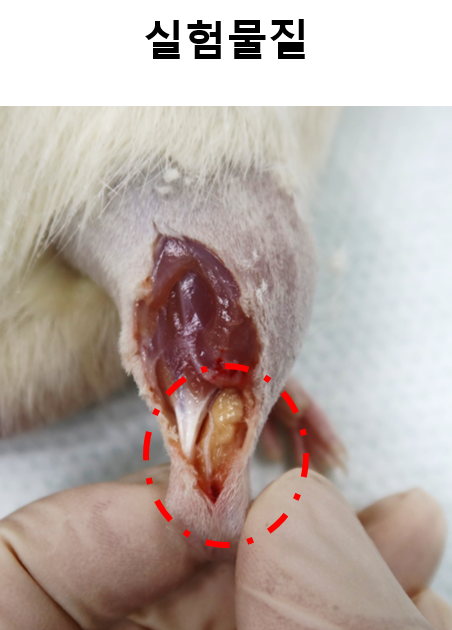


**Figure S7. In situ retention of injectable DHT at 8 weeks post-implantation.** Representative gross images obtained at sacrifice at 8 weeks post-implantation demonstrate localized retention of the injectable DHT within the treated Achilles tendon region.
